# Tobacco counseling in the setting of thyroid eye disease

**DOI:** 10.5935/0004-2749.20220003

**Published:** 2022

**Authors:** Michael Jacob Schatz, Brendan C. McGeehan, Maureen G. Maguire, César A. Briceño

**Affiliations:** 1 Perelman School of Medicine at the University of Pennsylvania, Philadelphia, PA, USA; 2 Department of Ophthalmology, University of Pennsylvania, Philadelphia, PA, USA

**Keywords:** Tobacco use, Counseling, Thyroid diseases, Eye diseases, Graves’ disease Eye diseases, Uso de tabaco, Aconselhamento, Doenças da glândula tireóide, Doença de Graves, Oftalmopatias

## Abstract

**Purpose:**

To collect data on the rate and efficacy of tobacco counseling sessions delivered by ophthalmologists under the setting of patients with thyroid eye disease.

**Methods:**

We analyzed the electronic medical records of a digital cohort of patients who visited ophthalmologists at the University of Pennsylvania Health System from 2012 to 2017 with reference to the International Classification of Disease (ICD) codes for Graves’ disease, thyrotoxic exophthalmos, and/or thyroid eye disease. Tobacco histories were recorded at the first and last ophthalmology office visits or the most temporally proximal encounter in packs/day (ppd), and each ophthalmology visit note was analyzed to validate the occurrence of tobacco counseling.

**Results:**

A total of 435 patients met our study inclusion criteria, of which 72 (16.6%) were active smokers at the time of their first visit. Only 57 (79.2%) of these active smokers had recorded smoking burdens, 34 (59.6%) of which received at least one form of recorded tobacco counseling session. Nine (26.5%) of the subjects who received tobacco counseling and 1 (4.3%) of those who did not have a recorded counseling, quit smoking (risk difference of 22.1%; 95% CI, [1.7%, 39.1%]; p=0.04). In addition, 17 (50.0%) of the subjects who received counseling and 7 (30.4%) of those who did not have a recorded counseling, reduced their ppd consumption (risk difference of 19.6%; 95% CI [-6.3%, 41.3%]; p=0.18). Overall, 14 (25.5%) out of the 55 ophthalmologists who were active smokers had recorded evidence of tobacco counseling.

**Conclusions:**

Our cumulative results provide the consequence of both missed opportunities for tobacco counseling as well as its efficacy in the setting of thyroid eye disease.

## Abstract (pt)

**Objetivo:**

Fornecer informações sobre a ocorrência e a eficácia do aconselhamento sobre o uso de tabaco por oftalmologistas a pacientes com doenças oculares associadas à tireoide.

**Métodos:**

Analisamos os prontuários médicos eletrônicos de uma coorte digital de pacientes atendidos por oftalmologistas no Sistema de Saúde da Universidade da Pensilvânia entre o início de 2012 e o final de 2017 com os códigos da Classificação Internacional de Doenças (CID) para a doença de Graves, exoftalmia tireotóxica ou doença ocular associada à tireoide. Os históricos de uso de tabaco foram registrados na primeira e na última visita ao consultório de Oftalmologia, ou na visita mais próxima no tempo. A quantidade de maços/dia (mpd) e todas as anotações feitas nas visitas ao consultório de Oftalmologia foram analisadas para aconselhamento sobre o uso de tabaco.

**Resultados:**

Um total de 435 indivíduos preencheram os critérios de inclusão, dos quais 72 (16,6%) estavam fumando ativamente no momento do primeiro encontro. Apenas 57 (79,2%) desses indivíduos que fumam ativamente registraram queixas relacionadas ao tabagismo, sendo que 34 (59,6%) deles receberam alguma forma de aconselhamento sobre o uso de tabaco. Ao todo, 9 (26,5%) indivíduos dentre os que receberam aconselhamento sobre tabaco e 1 (4,3%) que não teve aconselhamento registrado pararam de fumar (diferença de risco de 22,1%; IC 95%, [1,7%, 39,1%]; *p*=0,04). Dentre aqueles que receberam aconselhamento, 17 (50,0%) reduziram seus mpd, além de 7 (30,4%) daqueles que não tiveram aconselhamento (diferença de risco de 19,6%; IC 95% [-6,3%, 41,3%]; *p*=0,18). No geral, 14 (25,5%) dos 55 oftalmologistas que tiveram um paciente fumante ativo registraram evidências de aconselhamento sobre o uso de tabaco.

**Conclusões:**

Os resultados deste estudo revelam tanto as oportunidades perdidas de aconselhamento sobre o uso do tabaco quanto a eficácia do aconselhamento no contexto de doenças oculares associadas à tireoide.

## INTRODUCTION

Smoking remains one of the greatest risk factors contributing to premature death and affecting virtually every organ system in the human body. There is a lack of immediacy of health consequences, such as hyp ertension and atherosclerosis, from tobacco consumption. Therefore, one of the challenges in tobacco counseling is the development of chronic diseases whose impacts do not clinically manifest until several years of toxic accumulation. A 2005 cross-sectional analysis suggested that people would be equally motivated to quit smoking out of the fear of developing blindness as they would from the fear of developing lung cancer, heart disease, or stroke^(1)^. In young people, however, the fear of blindness may motivate smoking cessation even more than the potential development of lung and heart diseases^(2)^.

Thyroid eye disease (TED) presents a unique opportunity for tobacco counseling considering the immediate threat of visual impairment throughout the disease course as a complication secondary to exposure keratopathy, orbital congestion, and optic nerve compression. Based on the knowledge about the inflammatory nature of cigarette smoke, it is a part of the standard of care to encourage smoking cessation in TED patients in addition to providing the conventional medical management to further reduce the development of inflammation (steroids ± adjunctive radiotherapy) and the possible surgical interventions (such as tarsorrhaphy and, in severe cases, orbital decompression)^(3)^. Smoking has been shown to not only increase the risk of developing TED but also to reduce the response to orbital radiotherapy and to oral^(4)^ and intravenous^(5)^ glucocorticoids. Moreover, smoking is known to delay the response to treatment of TED in a dose-dependent manner^(6)^. Despite the risk that smoking poses for patients with TED as well as the development of age-related macular degeneration or cataracts, a survey of over 3,000 Americans revealed that only 9.5% of the patients were aware that smoking could cause blindness^(7)^. Advising cessation of smoking and conducting frequent follow-up on an outpatient basis by physicians can increase the 1-year smoking cessation rates from 0.1% to 5%^(8)^. A 2002 survey revealed that only 30% and 16% of ophthalmologists and optometrists, respectively, regularly advised their patients to quit smoking. The most concerning barriers to smoking cessation counseling recorded by this survey analysis was the lack of time for ophthalmologists and the lack of patient materials or training for optometrists^(9)^.

In contrast to other eye diseases that are complicated by smoking, TED has an active inflammatory phase that makes tobacco counseling urgently imperative. However, until date, there is no published literature on how frequently ophthalmologists should offer tobacco counseling to patients or on the success of such counseling sessions under a specific setting of TED.

## METHODS

The Institutional Review Board (IRB) approval was obtained, and the exemption status was awarded to the study. All stages of this research were performed in compliance with the Health Insurance Portability and Accountability Act and they adhered to the tenets of the Declaration of Helsinki as amended in 2008. A patient cohort was compiled by using the Penn Data Analytics’ PennSeek search tool. This tool allows researchers to scan unstructured and semi-structured text within the electronic medical records (PennChart) of the University of Pennsylvania Health System. However, it also included imported documents from the external health systems. The inclusion criteria for the study subjects were as follows: current age of 18-120 years; a contact date between 01/01/2012 and 12/31/2017; examination at the Scheie Ophthalmology Penn Presbyterian Medical Center, Scheie Procedures and Diagnostic Center Penn Presbyterian Medical Center, and/ or Scheie Eye Associates Hospital of the University of Pennsylvania; and a diagnosis code of E05.00 (ICD 10) and/or 242.00 (ICD 9). These codes of the International Classification of Diseases (ICD) are associated with a range of diseases and conditions, including Graves’ disease, thyrotoxic exophthalmos, toxic diffuse goiter, as well as the larger umbrella diagnosis of TED.

These search parameters were used to identify 447 patients whose medical records were securely exported. One of the authors (MJS) extracted data and recorded them securely. Of all patients, 12 were excluded from the analysis although they fulfilled the search criteria, because they did not fulfill the inclusion criterion of having made at least 1 office visit with an ophthalmologist, and 2 patients did not fulfill the inclusion criterion of having a clear smoking status.

The history of smoking (of cigarettes and cigars) and using smokeless tobacco (e.g., snuff or chew) in packs per day (ppd) were recorded from the time of each patient’s first and last ophthalmology office visits. If the smoking histories were not recorded at these visits, the next most temporally proximal clinical visit, not limited to any particular healthcare worker, was considered instead. Each patient’s general substance history was reviewed to determine whether the patient was a former smoker, and, if so, the time since quitting was noted. The total number of ophthalmology office visits as well as the type of tobacco counseling was recorded for each patient and for each physician involved in the care of any one of the patients with TED.

Each ophthalmology office visit note was scanned for the predetermined key phrases using the native PennChart search tools. These search phrases included “smoking,” “cigarettes,” “tobacco,” “thyroid,” “Graves,” as well as abbreviations of these terms. Using these search phrases, every ophthalmology office visit note was individually assessed to determine the patient’s tobacco burden and whether the ophthalmologist had recorded any tobacco counseling efforts.

We grouped tobacco counseling into 4 broad categories, as shown in table 1. Although the counseling sections within ophthalmologists’ office visit notes were identified using search phrases, the precise categorization of the counseling efforts was determined using a subjective qualitative assessment by the abstractor (MJS) based upon these predetermined definitions.

**Table 1:** Tobacco counseling categories

	Description	Example
Recommendation of anti-smoking medications	The patient is a current smoker and was offered some form medical-assisted therapies for quitting the use of tobacco products, including, but are not limited to varenicline (CHANTIX^®^, Pfizer, Mission, Kansas, USA), bupropion (Zyban^®^, GlaxoSmithKline, Philadelphia, PA, USA), and nicotine-replacement therapies.	A prescription for Chantix was included in the assessment and plan.
Referral to another provider for tobacco cessation	The patient is a current smoker and was referred to another healthcare professional for the purposes of facilitating quitting of the use of tobacco products.	“...will contact PCP re: smoking cessation strategies.”
Education on risk	The importance of avoiding tobacco products was explained to the patient.	“[T]he patient was counseled about deleterious effects of smoking and its implications in worsening of the eye disease.” “...the risk of blindness discussed.” “[Patient] instructed to avoid smoking.”
Written materials provided	The patient was provided written materials on the harmful effects of tobacco and/or tips for quitting.	“Written material on thyroid eye disease and adverse effects of smoking provided to the patient.”

The extracted data was aggregated and analyzed using a suite of tools and formulas available on the Microsoft Excel and Google Spreadsheets as well as the R version 3.5.1 software. All protected health information were converted into non-identifiable codes, and unique identification tags were used for the purposes of security.

The proportion of male versus female smokers was tested using a Chi-square test of 2 independent proportions. Due to the small sample size, Fisher’s exact test was selected to compare the association of counseling to the outcomes of quitting and reducing smoking. The confidence intervals for the corresponding risk difference were computed using the Newcombe’s method^(10)^.

## RESULTS

The characteristics of the 435 patients who met the study eligibility criteria are summarized in table 2. Of all, 70 (16.1%) patients were men and 365 (83.9%) were women. Most of the patients, 229 (52.6%) were recorded as “never smokers”. The smoking status at the initial visit was noted for 72 (16.6%) patients. The characteristics of the patients who were smoking at their initial visit are listed in table 2. No statistically significant difference was noted between the percent of men (12.9%) and women (17.3%) who were smokers (*p*=0.46). A total of 134 (30.8%) patients were recorded as having previously quit smoking. Of the 104 patients for whom the time of quitting was recorded, 14 (13.5%) had quit within the last year. Among 360 patients for whom the history of use of smokeless tobacco was recorded, 2 (0.6%) were active users and 10 (2.8%) had quit the use.

**Table 2:** Characteristics of patients

Characteristic	All patients			Patients smoking status at initial visit		
	Female (N=365)	Male (N=70)	Total (N=435)	Female (N=63)	Male (N=9)	Total (N=72)
Age (years)						
- Mean (SD)	52.1 (14.6)	53.5 (17.3)	52.4 (15.1)	52.2 (10.7)	48.0 (11.7)	51.6 (10.9)
- Median (Q1, Q3)	53 (43, 62)	55.5 (42, 64)	53 (43, 63)	52 (45.5, 57.5)	49 (40, 53)	51.5 (44.8, 57.2)
- Range	18-91	18-90	18-91	29-74	32-65	29-74
Race, n (%)						
- Asian	24 (6.6%)	5 (7.1%)	29 (6.7%)	0 (0.0%)	0 (0.0%)	0 (0.0%)
- Black	142 (38.9%)	7 (10.0%)	149 (34.3%)	35 (55.6%)	2 (22.2%)	37 (51.4%)
- Other	32 (8.8%)	11 (15.7%)	43 (9.9%)	2 (3.2%)	3 (33.3%)	5 (6.9%)
- White	167 (45.8%)	47 (67.1%)	214 (49.2%)	26 (41.3%)	4 (44.4%)	30 (41.7%)
Ethnicity, n (%)						
- Hispanic	6 (1.6%)	4 (5.7%)	10 (2.3%)	1 (1.6%)	2 (22.2%)	3 (4.2%)
- Non-Hispanic	358 (98.1%)	66 (94.3%)	424 (97.5%)	62 (98.4%)	7 (77.8%)	69 (95.8%)
- Unknown	1 (0.3%)	0 (0.0%)	1 (0.2%)	0 (0.0%)	0 (0.0%)	0 (0.0%)
Language, n (%)						
- English	355 (97.3%)	64 (91.4%)	419 (96.3%)	62 (98.4%)	7 (77.8%)	69 (95.8%)
- Other	10 (2.7%)	6 (8.6%)	16 (3.7%)	1 (1.6%)	2 (22.2%)	3 (4.2%)
Interpreter, n (%)						
- No	360 (98.6%)	67 (95.7%)	427 (98.2%)	62 (98.4%)	8 (88.9%)	70 (97.2%)
- Yes	5 (1.4%)	3 (4.3%)	8 (1.8%)	1 (1.6%)	1 (11.1%)	2 (2.8%)
Initial Smoking Burden (packs per day)						
- N-Missing	53	8	61	14	0	14^*^
- Mean (SD)	0.11 (0.30)	0.09 (0.26)	0.10 (0.30)	0.66 (0.45)	0.61 (0.40)	0.65 (0.44)
- Median (Q1, Q3)	0.00 (0.00, 0.00)	0.00 (0.00, 0.00)	0.00 (0.00, 0.00)	0.50 (0.50, 1.00)	0.50 (0.50, 0.50)	0.50 (0.50, 1.00)
- Range	0.00-2.00	0.00-1.50	0.00-2.00	0.025-2.0	0.20-1.5	0.025-2.0
Follow-up time (years)						
- N-Missing	51	10	61	10	2	12
- Mean (SD)	2.8 (3.0)	1.9 (2.5)	2.6 (2.9)	2.4 (2.9)	1.6 (1.8)	2.3 (2.8)
- Median (Q1, Q3)	1.7 (0.2, 4.7)	0.9 (0.0, 2.4)	1.5 (0.1, 4.4)	1.4 (0, 4.0)	0.8 (0.4, 2.3)	1.3 (0.0, 3.9)

* There were only 14 patients with missing initial smoking burdens, but a total of 15 patients had missing initial and/or final smoking burdens.

A summary of the types of smoking cessation counseling offered to the study subjects is provided in table 3. Tobacco counseling was offered at one or more visits to 34 (59.6%) of the active smokers; in all cases, the counseling was offered in the form of an education of the risk with a few other forms of tobacco counseling delivered. Tobacco counseling in the form of education on the risk was offered to 31 (23.1%) of former smokers and 32 (14%) of never smokers.

**Table 3:** Tobacco counseling offered to patients by smoking status

Any ophthalmology office visit with	Smoking status					
	Current (N=72) n (%)		Former (N=134) n (%)		Never (N=229) n (%)	
Recommendation of anti-smoking medications	1	(1.4)	0	(0.0)	0	(0.0)
Referral to another provider for tobacco cessation	2	(2.8)	0	(0.0)	0	(0.0)
Education on risk	36	(50.0)	31	(23.1)	32	(14.0)
Written materials provided	4	(5.6)	1	(0.8)	0	(0.0)
Any of the above^**^	36	(50.0)	31	(23.1)	32	(14.0)

** This reflects the number of patients with at least one of any kind of tobacco counseling efforts. It is not the sum of other categories due to an overlap.

Of the 72 patients who were smokers at the time of their first ophthalmology office visit, 57 had smoking burdens recorded for both their first and last ophthalmology visits. The changes in the smoking burden from their first to the last visits with an ophthalmologist have been summarized in table 4. Overall, 10 (17.5%) of the subjects quit smoking and an additional 24 (42.1%) reduced the number of ppd consumed. Among the 34 patients for whom recorded evidence of tobacco counseling was present, 9 (26.5%) quit smoking and 17 (50.0%) reduced their ppd consumption. Among the 23 patients with no recorded counseling, only 1 (4.3%) quit and 7 (30.4%) reduced their ppd consumption, which yielded a difference of 22.1% (95% CI, [1.7%, 39.1%]; p=0.04) for quitting and a difference of 19.6% (95% CI [-6.3%, 41.3%]; p=0.18) in the reduction of ppd consumption.

**Table 4:** Change in tobacco burden for smoking patients between first and last visits by receipt of tobacco counseling

	Patients initially smoking		Received tobacco counseling	
**Change in smoking**	**All (N= 57) n (%)**		**Yes (N=34) n (%)**	**No (N=23) n (%)**
Quit smoking	10	(17.5)	9 (26.5)	1 (4.3)
Reduction in packs per day	24	(42.1)	17 (50.0)	7 (30.4)
No change	31	(54.4)	0 (0.0)	2 (8.7)
Increase in packs per day	2	(3.5)	17 (50.0)	14 (60.9)

A total of 111 ophthalmologists who had one or more visits of at least one of the study patients, represented nearly 4,000 unique ophthalmologist office visit notes, of these 55 ophthalmologists had at least one office visit by an active smoker patient. The efforts of these ophthalmologists in tobacco counseling are summarized in table 5. Of all, 14 (25.5%) ophthalmologists presented recorded evidence of tobacco counseling sessions at one or more patient visits.

**Table 5:** Ophthalmologists (N=55) offering tobacco counseling to active smokers

Any ophthalmology office visit with	Ophthalmologists (N=55) n (%)
Recommendation of anti-smoking medications	1 (1.8)
Referral to another provider for tobacco cessation	3 (5.5)
Education on risk	14 (25.5)
Written materials provided	1 (1.8)
Any of the above	14 (25.5)

## DISCUSSION

Having at least one recorded session on tobacco counseling by an ophthalmologist increased the probability that an active smoker would either quit smoking altogether or reduce their consumption substantially. Although both the statistical outcomes showed large confidence intervals, the risk difference in the smoking quitting rates did rise to the level of 5% statistical significance. We therefore believe that future studies should involve a larger patient cohort to better power these analyses.

The female: male ratio in our cohort was 5.21, which is slightly larger than a previously reported ratio of 4.05^(11)^. The active smoking rates among the female and male subjects in our cohort was 17.3% and 12.9%, respectively, which is an intermediate value between national averages (women: 12.0%, men: 15.6%)^(12)^ and the prior epidemiology studies on patients with Graves disease (women: 27.5%, men: 31.4%)^(13)^.

The quitting rate among the active smokers in our cohort was quite high at 17.5%, but it was even higher (26.5%) when they received at least one form of documented tobacco counseling session by an ophthalmologist. For comparison purpose, one randomly controlled trial revealed that the 6-month sustained smoking abstinence rates was only 6.0% for patients who received standard informational resources and, in some cases, access to nicotine-replacement therapies; and it was 9.4-16.0% for various financial reward-based incentive programs^(14)^. One explanation for the high cessation rates in the present study is that the smoking status was determined based on the documentation in office visit notes, which in turn was dependent on patient communication. We suggest that the patients could be incentivized to downplay their smoking use in order to avoid judgment by healthcare workers. Future studies could remedy this situation by regularly undertaking biochemical tests to quantifiably determine the sustained smoking abstinence rates.

Moreover, 74.5% of the ophthalmologists with at least one office visit by an active smoker recorded zero incidents of using any recorded form of tobacco counseling. Importantly, the absence of documented tobacco counseling session does not indicate that no counseling happened, considering that an ophthalmologist may verbally counsel a patient but forget to document it. Since documentation of tobacco counseling efforts afford some billing benefits, it was believed that it could be used as a reasonable proxy for verbal communication. Moreover, the documentation of tobacco counseling provides, although imperfect, some sense of the significance of such efforts in an ophthalmologist’s assessment and plan, thereby representing a valuable metric independent of verbal counseling.

Dissemination of reading and video materials as well as grand rounds lectures by addiction medicine specialist guest speakers are only a few strategies to increase both the adoption and efficacy of tobacco counseling by ophthalmologists. Future studies should therefore explore options of training ophthalmologists on nicotine replacement and medication-assisted therapies.

Tobacco counseling is already universally considered as the standard of practice for nearly every medical specialty and not just for ophthalmologists in the setting of new patients with TED. Tobacco counseling is free and can be performed in a few seconds; imparting such counseling has been associated with reductions in the nicotine burden both in the literature as well as in the present study. Therefore, future studies should focus on ways to increase the rate of delivering tobacco counseling among ophthalmologists and to improve the efficacy of the said counseling.
